# DIFFERENCES BETWEEN TRAINEE AND SUPERVISOR PERSPECTIVES ON THE IMPACT OF COVID-19 ON GEROPSYCHOLOGY TRAINING

**DOI:** 10.1093/geroni/igad104.3278

**Published:** 2023-12-21

**Authors:** Valerie Luskey, M Lindsey Jacobs, Michelle Mlinac, Hannah Bashian, Candice Reel, Julia Boyle

**Affiliations:** University of Alabama, Tuscaloosa, Alabama, United States; University of Alabama, Tuscaloosa, Alabama, United States; VA Boston Healthcare System, Boston, Massachusetts, United States; VA Boston Healthcare System, Boston, Massachusetts, United States; University of Alabama, Tuscaloosa, Alabama, United States; VA Boston Healthcare System, Boston, Massachusetts, United States

## Abstract

The COVID-19 pandemic majorly impacted geriatric specialty training. To better understand this impact, a survey study was conducted to examine the pandemic’s unique impact on geropsychology training. Participants (N=62) included psychology trainees specializing in geropsychology and psychologists providing geropsychology training. The majority of participants identified as white (90.3%), female (87.1%), and 30-39 years old. Using a 5-point Likert scale ranging from “not at all impactful” to “extremely impactful”, participants rated their subjective experience on how the pandemic positively and negatively affected geropsychology training in three functional competency areas (assessment, intervention, and consultation) consistent with the Pikes Peak Model for Geropsychology Training. A 2x3 mixed design repeated measures ANOVA was conducted to examine similarities and differences in trainees’ and supervisors’ perceptions of the pandemic’s impact on the competency domains. Results indicate that supervisors’ and trainees’ perception of the pandemic’s negative impacts aligned, F(1,43)=.01, p=.931. Results were consistent across all competency domains, F(2,86)=1.39, p=.26; however, perceptions of the positive impacts significantly differed, F(2,84)=6.459, p=.002. Overall, both trainees and supervisors perceived more positive impacts on intervention and consultation compared to assessment, t(43)=3.54, p≤.001. However, trainees (M=1.8, SD=.112) reported more positive impacts overall compared to supervisors (M=1.3, SD=.09), F(1,42)=9.08, p=.004. These findings have significant implications for the level of competence in emerging geropsychologists’, particularly in the areas of cognitive and capacity assessments. Recommendations for additional assessment training and supervision will be provided.

